# Clinical Characteristics and Outcomes of Catheter Ablation in Young Patients With Atrial Fibrillation

**DOI:** 10.1002/clc.70144

**Published:** 2025-05-13

**Authors:** Xuewen Wang, Qiqi Cao, Tao Liu, Fan Zhang, Shujuan Zhang, Shaobo Shi, Qingyan Zhao, He Huang, Congxin Huang

**Affiliations:** ^1^ Cardiovascular Department Renmin Hospital of Wuhan University Wuhan Hubei China; ^2^ Cardiovascular Research Institute Wuhan University Wuhan Hubei China; ^3^ Hubei Key Laboratory of Cardiology Wuhan University Wuhan Hubei China; ^4^ Cardiovascular Department, Taihe Hospital Hubei University of Medicine Shiyan Hubei China

**Keywords:** atrial fibrillation, catheter ablation, clinical characteristic, young people

## Abstract

**Background:**

Data concerning young patients with atrial fibrillation (AF) are currently limited.

**Hypothesis:**

This study aimed to assess the clinical characteristics and risk factors for AF recurrence in young patients following catheter ablation (CA).

**Methods:**

All AF patients aged ≤ 45 years who underwent CA were identified from the China Atrial Fibrillation Center database between September 2018 and September 2023. Baseline clinical characteristics, procedural details, and follow‐up outcomes were compared between the paroxysmal and non‐paroxysmal cohorts.

**Results:**

A total of 6,531 young patients with AF were included in the final analysis, with an average age of 37.7 ± 5.5 years, 77.1% were male, and 65.2% with paroxysmal AF. The primary comorbidities were hypertension (16.2%), heart failure (8.4%), valvular heart disease (3.5%), diabetes mellitus (3.2%), peripheral arterial disease (2.8%), stroke/transient ischemic attack (2.5%), and cardiomyopathy (2.2%). Following CA, the recurrence rate of AF post‐CA was 14.4% across the entire cohort, with a lower recurrence rate in the paroxysmal AF group compared to the non‐paroxysmal AF group (10.6% vs. 19.7%, *p* < 0.001). Non‐paroxysmal AF (HR 2.34, 95% CI 1.62 to 3.36, *p* < 0.001) and hypertension (HR 1.69, 95% CI 1.09 to 2.63, *p* = 0.019) were identified as independent predictors for AF recurrence.

**Conclusion:**

Young AF patients who undergo CA exhibit a low recurrence rate and a notable improvement in symptoms post‐CA. Non‐paroxysmal AF and hypertension emerge as primary contributors to AF recurrence following CA in this population.

## Graphical abstract

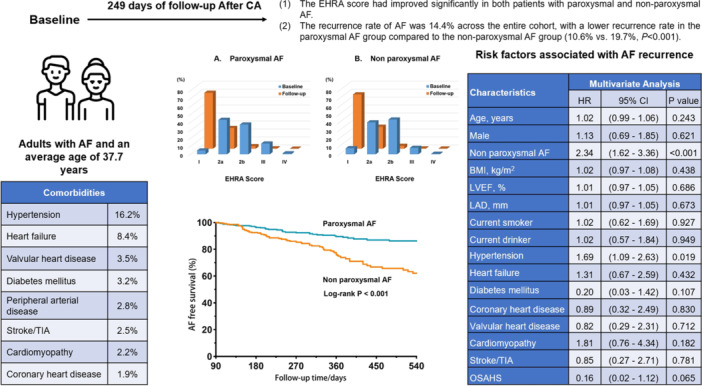

## 1 Introduction

Atrial fibrillation (AF) is the most prevalent cardiac arrhythmia. In China, the age‐standardized prevalence of AF stands at 1.6%. Utilizing data from the sixth national census, it is estimated that approximately 20 million individuals are affected by this condition [1]. Catheter ablation (CA) has emerged as a well‐established approach for restoring sinus rhythm and symptom improvement among AF patients [2, 3]. Nonetheless, its efficacy is not uniformly satisfactory. Prior research indicated that factors such as left atrium size, duration of AF, age, and various clinical comorbidities significantly influence the recurrence rate of AF post‐CA [3]. While most existing research has concentrated on older patients, evidence specific to young patients remains limited [4, 5]. Despite advanced age being recognized as a paramount risk factor for AF development, there has been a notable rise in the prevalence of young‐onset AF in recent years [6]. Notably, CA has demonstrated enhanced safety and efficacy outcomes in young patients compared to older patients [7, 8]. Previous clinical investigations have highlighted structural heart disease as the only independent predictor for AF recurrence post‐CA among young patients [7]. However, structural heart disease is not prevalent among young AF patients, hence, the recurrence rate and potential risk factors for young patients remain unclear. This study aims to further investigate this issue.

## 2 Methods

### 2.1 Patients and Study Design

Patients with AF aged ≤ 45 years who underwent a single CA were identified from the China Atrial Fibrillation Center database (https://ky.china-afc.org) between September 2018 and September 2023. Patients with repeated admissions, repeated CA, or those who did not undergo CA were excluded. Catheter ablation includes radiofrequency ablation and cryoablation, but excludes pulsed field ablation and surgical ablation. The study was a component of the real‐world study of Chinese atrial fibrillation, which comprising 650 standard atrial fibrillation centers and 282 basic atrial fibrillation centers. The study aims to collect data on atrial fibrillation screening, outpatient visits, hospitalizations, surgeries, and follow‐ups from each unit to assess the real‐time management capabilities of these centers. The evaluation is based on the guidelines for the management of atrial fibrillation, with the results ultimately fed back to the centers to enhance the overall atrial fibrillation management level in China. Data from medical records were gathered using a standardized electronic form system. In each participating hospital, patients were followed up through telephone interviews or outpatient visits three months after discharge. The subjects under investigation are divided into paroxysmal AF group and non‐paroxysmal AF group, with an analysis on the clinical and CA characteristics, as well as follow‐up outcomes. We adhered to the STROBE statement for reporting observational studies. The registry is being conducted in accordance with the principles of the Declaration of Helsinki, local regulatory requirements, and clinical practice guidelines (registration number: ChiCTR1900021250). This study was approved by the Institutional Committee on Human Research at Renmin Hospital of Wuhan University (Wuhan, China). Written informed consent was waived due to retrospective analysis of electronic medical records, and the patient private information was deidentified.

### 2.2 Data Collection and Definitions

Data on clinical characteristics, procedure features, and follow‐up were collected from the electronic records system. Clinical variables encompassed demographics (age and sex), anthropometrics (body weight and height), symptoms, and vital signs (blood pressure and heart rate) at admission, AF pattern, CHA_2_DS_2_‐VASc scores, smoking and drinking habits, comorbidities (hypertension [HTN], heart failure [HF], valvular heart disease, diabetes mellitus [DM], peripheral arterial disease [PAD], prior stroke/transient ischemic attack [TIA], cardiomyopathy, coronary heart disease [CHD], thyroid disease, and obstructive sleep apnea‐hypopnea syndrome [OSAHS]), echocardiography results, and treatment modalities. Procedure‐related parameters included anesthesia type, pre‐ and postablation heart rhythm, heparin dosage, activated clotting time (ACT), X‐ray fluoroscopy usage, ablation strategies, ablation endpoint, electrical cardioversion, verification method, and procedural complications. The European Heart Rhythm Association (EHRA) symptom score was employed to assess AF patients’ symptoms at baseline and during follow‐up.

According to the 2021 European Society of Cardiology (ESC) Guidelines, AF was defined as a standard 12‐lead electrocardiogram (ECG) recording or a single‐lead ECG tracing of ≥ 30 s showing a heart rhythm devoid of discernible repeating P waves and irregular RR intervals, or a documented medical history of diagnosed AF [3]. Paroxysmal AF refers to episodes terminating within ≤ 7 days of onset, while non‐paroxysmal AF encompasses persistent AF, long‐standing persistent AF, and permanent AF. AF recurrence was defined as an episode of AF, atrial flutter, or atrial tachycardia lasting ≥ 30 s confirmed by ECG or 24‐h Holter monitoring after a 3‐months blanking period postablation procedure [3, 9]. Body mass index (BMI) was calculated as weight divided by the square of height (kg/m^2^).

### 2.3 Statistical Analysis

Data analysis was conducted using SPSS 26 software (IBM, West Grove, Pennsylvania, USA). Continuous variables were expressed as mean ± standard deviation (SD) or median and interquartile range (IQR), categorical variables were presented as counts and percentages (%). Group comparisons were performed using 2‐sample *t* tests or Kruskal–Wallis tests for continuous variables, and Chi‐square tests or Fisher exact tests for categorical variables, as deemed appropriate. Predictors of AF recurrence post‐CA were determined using a Cox regression model. *p* < 0.05 was considered statistically significant.

## 3 Results

### 3.1 Clinical Characteristics

**Figure 1:**
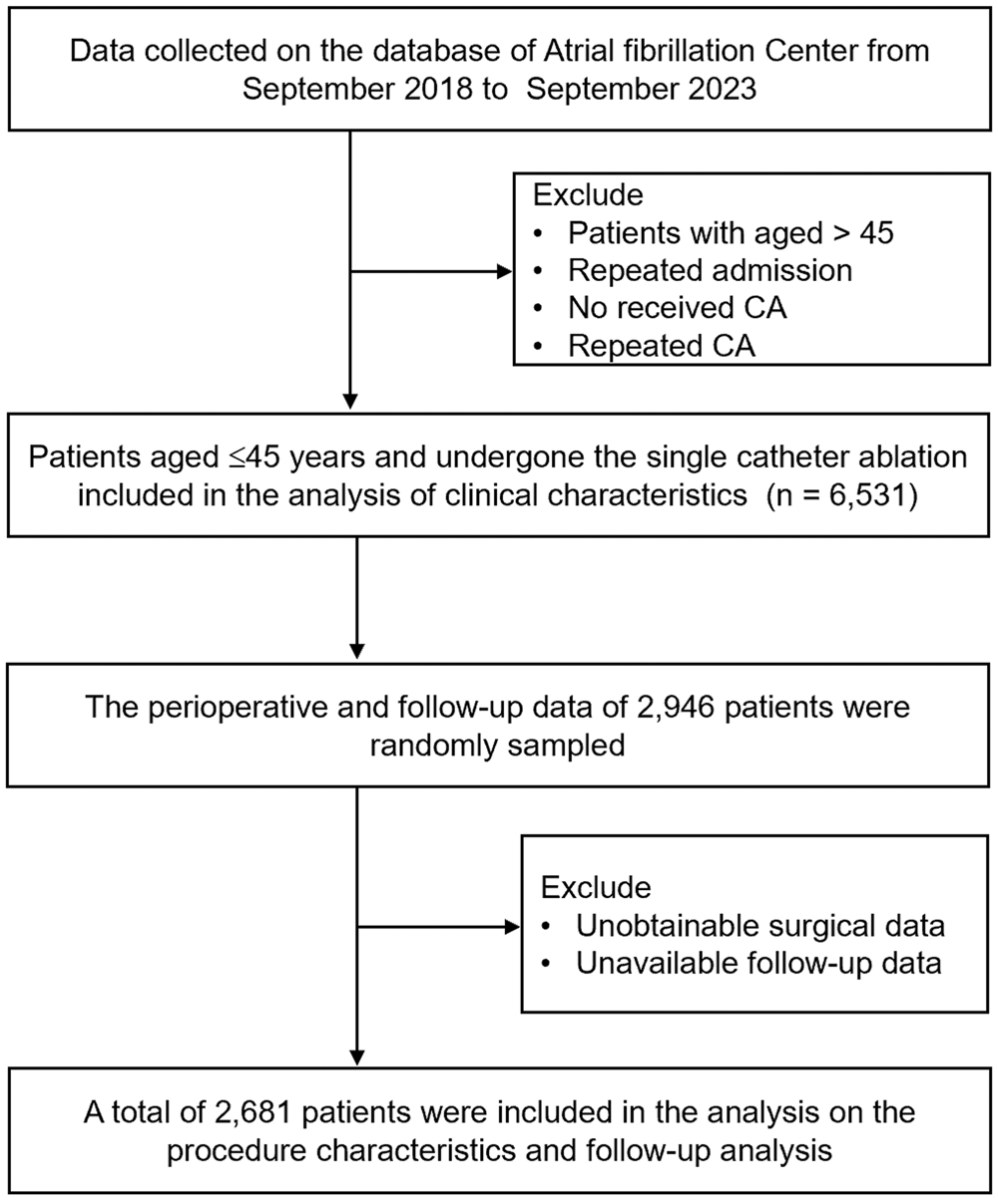
Study flowchart.

Between September 2018 and September 2023, following the exclusion of patients who did not meet the criteria, a total of 6,531 patients were ultimately included in the analysis of clinical characteristics in the registry (Figure 1). Among these patients, with an average age of 37.7 ± 5.5 years, 77.1% were male, and 65.2% had paroxysmal AF. The baseline clinical characteristics are detailed in Table 1. Compared to patients with non‐paroxysmal AF, those with paroxysmal AF were more likely to be younger, lower BMI, less percentage of asymptomatic cases, lower systolic blood pressure, slower heart rate, higher left ventricular ejection fraction (LVEF), smaller left atrial diameter (LAD), and have shorter hospital stays. Conversely, patients with non‐paroxysmal AF had a higher incidence of HF, valvular heart disease, PAD, stroke/TIA, and cardiomyopathy than those with paroxysmal AF. The most commonly used antiarrhythmic drugs were amiodarone/dronedarone (29.5%), with a significant difference between paroxysmal AF and non‐paroxysmal AF (10.5% vs. 34.5%, *p* < 0.001), followed by β‐blockers (26.2% vs. 22.3%, *p* = 0.001). Patients with non‐paroxysmal AF also had a higher proportion of electrical cardioversion, traditional Chinese medicines, and vitamin K antagonists use compared to those with paroxysmal AF (all *p* < 0.001).

**Table 1:** The baseline clinical characteristics of young patients with atrial fibrillation.

Characteristics	Total (*n* = 6531)	Paroxysmal AF (*n* = 4256)	Non paroxysmal AF (*n* = 2275)	*P* value
Age, years	37.7 ± 5.5	37.4 ± 5.6	38.2 ± 5.3	< 0.001
Gender, male	5034 (77.1)	3263 (76.7)	1771 (77.8)	0.281
BMI, kg/m^2^	25.0 ± 3.7	24.9 ± 3.6	25.2 ± 3.9	0.005
Hospital stays, days	6.6 ± 2.9	6.4 ± 2.8	6.9 ± 3.0	< 0.001
Asymptomatic	187 (2.9)	99 (2.3)	88 (3.9)	<0.001
CHA_2_DS_2_‐VASc score				
0	4707 (72.1)	3121 (73.3)	1586 (69.7)	0.002
1	1279 (19.6)	844 (19.8)	435 (19.1)	0.491
≥ 2	545 (8.3)	291 (6.8)	254 (11.2)	< 0.001
Current smoker	754 (11.5)	508 (11.9)	246 (10.8)	0.176
Current drinker	346 (5.3)	221 (5.2)	125 (5.5)	0.604
Comorbidities				
HTN	1060 (16.2)	722 (17.0)	338 (14.9)	0.028
HF	550 (8.4)	251 (5.9)	299 (13.1)	< 0.001
Valvular heart disease	229 (3.5)	73 (1.7)	156 (6.9)	< 0.001
DM	212 (3.2)	137 (3.2)	75 (3.3)	0.866
PAD	182 (2.8)	104 (2.4)	78 (3.4)	0.021
Stroke/TIA	161 (2.5)	81 (1.9)	80 (3.5)	< 0.001
Cardiomyopathy	142 (2.2)	61 (1.4)	81 (3.6)	< 0.001
CHD	126 (1.9)	90 (2.1)	36 (1.6)	0.136
Thyroid disease	106 (1.6)	61 (1.4)	45 (2.0)	0.097
OSAHS	61 (0.9)	29 (0.7)	22 (1.0)	0.211
Systolic BP, mmHg	125 ± 14	124 ± 14	127 ± 13	< 0.001
Heart rate, beats per min	84 ± 15	81 ± 15	89 ± 14	< 0.001
LVEF, %	60 ± 5	61 ± 4	58 ± 5	< 0.001
LAD, mm	37 ± 5	35 ± 4	40 ± 4	< 0.001
CIEDs	11 (0.2)	6 (0.1)	5 (0.2)	0.459
Electrical cardioversion	209 (3.2)	90 (2.1)	119 (5.2)	<0.001
Antiarrhythmic drugs				
Amiodarone/dronedarone	1230 (18.8)	446 (10.5)	784 (34.5)	< 0.001
β receptor blockers	1621 (24.8)	1114 (26.2)	507 (22.3)	0.001
Propafenone	414 (6.3)	340 (8.0)	74 (3.3)	< 0.001
Traditional Chinese medicines	186 (2.8)	82 (1.9)	104 (4.6)	< 0.001
CCBs	56 (0.9)	34 (0.8)	22 (1.0)	0.483
Anticoagulants				
NOACs	1116 (17.1)	745 (17.5)	371 (16.3)	0.221
VitK antagonists	197 (3.0)	85 (2.0)	112 (4.9)	< 0.001

*Notes:* variables present as *n* (%), mean ± SD, or median (IQR).

Abbreviations: AF, atrial fibrillation; BMI, body mass index; BP, blood pressure; CHD, coronary heart disease; CIEDs, cardiac implantable electronic devices; DM, diabetes mellites; HF, heart failure; HTN, hypertension; LAD, left atrial diameter; LVEF, left ventricular ejection fraction; NOACs, Novel oral anticoagulants; OSAHS, obstructive sleep apnea‐hypopnea syndrome; PAD, peripheral arterial disease; TIA, transient ischemic attack.

### 3.2 AF Ablation and Recurrence

A total of 2681 patients who completed follow‐up and were included in the analysis of procedure features and outcomes following CA for AF. Overall, 73.4% of the patients had paroxysmal AF, 16.1% received general anesthesia for ablation, 43.9% of patients had AF rhythm before the procedure, 96.6% of patients returned to sinus rhythm after the procedure, almost all patients (99.5%) underwent pulmonary venous isolation (PVI), and 33.1% underwent linear ablation. Procedure complications occurred in 25 (0.9%) patients (Table 2).

**Table 2:** Clinical characteristics during the perioperative period of catheter ablation.

Characteristics	Total (*n* = 2681)	Paroxysmal AF (*n* = 1968)	Non paroxysmal AF (*n* = 713)	*P* value
General anesthesia	431 (16.1)	271 (13.8)	160 (22.4)	< 0.01
Rhythm before ablation				
SR	1297 (48.4)	1251 (63.6)	46 (6.5)	< 0.001
AF	1176 (43.9)	548 (27.8)	628 (88.1)	
Other	208 (7.8)	169 (8.6)	39 (5.5)	
Rhythm after ablation				
SR	2591 (96.6)	1908 (97.0)	683 (95.8)	0.143
AF	34 (1.3)	2 (1.0)	14 (2.0)	
Other	56 (2.1)	40 (2.0)	16 (2.2)	
Heparin, U	7000 (6000–9000)	7000 (6000–9000)	8000 (6200–9800)	< 0.001
ACT				
Median, seconds	256 (222–300)	253 (221–300)	300 (251–340)	0.003
No detection	1539 (57.4)	1,139 (57.9)	400 (56.1)	0.412
X‐ray fluoroscopy				
Fluoroscopy time, min	16.5 ± 10.6	16.6 ± 10.9	16.5 ± 9.8	0.816
Radiation dose, mGy	150 (66–267)	150 (66–250)	150 (66–300)	0.139
Procedure duration, min	152 ± 50	148 ± 45	168 ± 55	< 0.001
Ablation strategies				
PVI	2667 (99.5)	1954 (99.3)	713 (100.0)	0.024
Linear lesions	887 (33.1)	497 (25.3)	390 (54.7)	< 0.001
LA roof	391 (14.6)	123 (6.3)	268 (37.6)	< 0.001
LA isthmus	246 (9.2)	97 (4.9)	149 (20.9)	< 0.001
LA posterior	116 (4.3)	45 (2.3)	71 (10.0)	< 0.001
RA isthmus	319 (11.9)	208 (10.6)	111 (15.6)	< 0.001
SVC isolation	14 (5.5)	122 (6.2)	26 (3.6)	0.011
CFAEs	55 (2.1)	30 (1.5)	25 (3.5)	0.001
Rotors	5 (0.2)	2 (0.1)	3 (0.4)	0.121
GP ablation	2 (0.1)	1 (0.1)	1 (0.1)	0.461
Other	90 (3.4)	67 (3.4)	23 (3.2)	0.82
Ablation endpoint				
PV potential disappears	2133 (79.6)	1558 (79.2)	575 (80.6)	0.402
Completion of linear lesions	1218 (45.4)	793 (40.3)	425 (59.6)	< 0.001
Bidirectional block	1210 (45.1)	783 (39.8)	338 (47.4)	< 0.001
Restore SR	1121 (41.8)	892 (45.3)	318 (44.6)	0.739
AF cannot be triggered	769 (28.7)	612 (31.1)	157 (22.0)	< 0.001
Electrical cardioversion	510 (19.0)	148 (7.5)	362 (50.8)	< 0.001
Provocative test				
Burst pacing	1260 (47.0)	367 (18.6)	101 (14.2)	0.007
Drugs	468 (17.5)	970 (49.3)	290 (40.7)	< 0.001
No performed	1246 (46.5)	860 (43.7)	386 (54.1)	< 0.001
Complications	25 (0.9)	18 (0.9)	7 (1.0)	0.873
Pericardial effusion	10 (0.4)	7 (0.51)	3 (0.4)	0.807
Cardiac tamponade	2 (0.1)	0 (0.0)	2 (0.3)	0.071
Vascular complications	3 (0.1)	3 (0.2)	0 (0.0)	0.570
Hematoma	3 (0.1)	3 (0.2)	0 (0.0)	0.570
Arteriovenous fistula	0 (0.0)	0 (0.0)	0 (0.0)	—
Pseudoaneurysm	0 (0.0)	0 (0.0)	0 (0.0)	—
Phrenic nerve palsy	1 (0.04)	1 (0.05)	0 (0.00)	1.000
Major bleeding	0 (0.0)	0 (0.0)	0 (0.0)	—
Thromboembolic event	0 (0.0)	0 (0.0)	0 (0.0)	—
Pulmonary vein stenosis	10 (0.4)	10 (0.5)	0 (0.00)	0.057
Esophageal perforation/fistula	2 (0.1)	0 (0.0)	2 (0.3)	0.019
Death	1 (0.04)	1 (0.05)	0 (0.00)	0.547
Other	6 (0.2)	4 (0.2)	2 (0.3)	0.660

*Notes:* variables present as *n* (%), mean ± SD, or median (IQR).

Abbreviations: ACT, activated coagulation time; AF, atrial fibrillation; CFAEs, complex fractionated electrograms; CTA, computed tomography angiography; GP, ganglionated plexi; LA, left atria; MRI, magnetic resonance imaging; PVI, pulmonary vein isolation; RA, right atria; SR, sinus rhythm; SVC, superior vena cava; TEE, transesophageal echocardiogram.

Compared to patients with paroxysmal AF, those with non‐paroxysmal AF exhibited a higher proportion of general anesthesia and pre‐CA AF rhythm, higher heparin dosage and mean ACT, and longer procedure duration. A total of 99.5% patients underwent PVI, with some also receiving linear lesions (paroxysmal AF: 25.3% vs. non‐paroxysmal AF: 54.7%, *p* < 0.001) and complex fractionated atrial electrograms (CFAEs) ablation (paroxysmal AF: 1.5% vs. non‐paroxysmal AF: 3.5%, *p* = 0.001), respectively. The ratio of superior vena cava isolation was higher in paroxysmal AF compared to non‐paroxysmal AF (6.2% vs. 3.6%, *p* = 0.011). However, the ratios of ganglionated plexi ablation and rotors ablation did not show significant differences between the two groups. At the end of the ablation, there was no significant differences in the disappearance of pulmonary vein potentials and the restoration of sinus rhythm between the two groups. However, the proportion of completion of linear lesions and bidirectional block was higher in non‐paroxysmal AF compared to paroxysmal AF (*p* < 0.001). Additionally, the proportion of postablation AF non‐inducibility was higher in paroxysmal AF compared to non‐paroxysmal AF (*p* < 0.001). Regarding ablation complications, there were two cases of esophageal fistula in the non‐paroxysmal AF group (*p* = 0.019) (Table 2).

**Figure 2:**
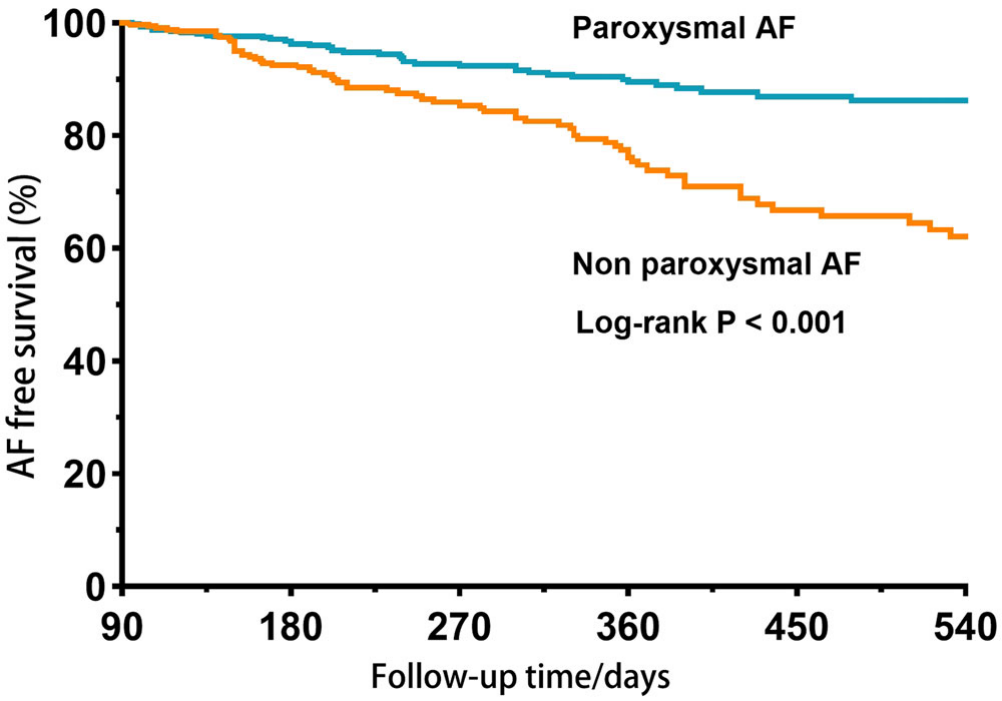
Kaplan‐Meier curve for AF free survival.

With a median follow‐up duration of 249 days, the overall recurrence rate of atrial tachyarrhythmia (AT) following a single ablation was 14.4%. Patients with paroxysmal AF exhibited a lower recurrence rate of AT compared to those with non‐paroxysmal AF (10.6% vs. 19.7%, *p* < 0.001) (Figure 2). Additionally, the symptoms of AF were significantly improved after CA in both groups (Figure S1). The univariate Cox regression model indicated that non‐paroxysmal AF (HR 2.39; 95% CI 1.68–3.42; *p* < 0.001), HTN (HR 1.75; 95% CI 1.16–2.62; *p* = 0.007), and cardiomyopathy (HR 2.53; 95% CI 1.11–5.77; *p* = 0.027) were associated with an increased risk of AF recurrence. Meanwhile, the multivariate Cox regression model identified non‐paroxysmal AF (HR 2.34; 95% CI 1.62–3.36; *p* < 0.001) and HTN (HR 1.69; 95% CI 1.09–2.63; *p* = 0.019) as independent risk factors for AF recurrence following CA in young patients (Table 3).

**Table 3:** Analysis of factors associated with atrial fibrillation recurrence following catheter ablation in young patients.

Characteristics	Univariate analysis			Multivariate analysis		
	HR	95% CI	*P* value	HR	95% CI	*P* value
Age, years	1.03	(1.00–1.07)	0.051	1.02	(0.99–1.06)	0.243
Male	1.16	(0.73–1.83)	0.539	1.13	(0.69–1.85)	0.621
Non paroxysmal AF	2.39	(1.68–3.42)	< 0.001	2.34	(1.62–3.36)	< 0.001
BMI, kg/m^2^	1.03	(0.98–1.08)	0.277	1.02	(0.97–1.08)	0.438
LVEF, %	1.01	(0.97–1.05)	0.771	1.01	(0.97–1.05)	0.686
LAD, mm	1.02	(0.98–1.05)	0.393	1.01	(0.97–1.05)	0.673
Current smoker	1.12	(0.73–1.73)	0.605	1.02	(0.62–1.69)	0.927
Current drinker	0.96	(0.57–1.60)	0.862	1.02	(0.57–1.84)	0.949
HTN	1.75	(1.16–2.62)	0.007	1.69	(1.09–2.63)	0.019
HF	1.51	(0.81–2.81)	0.191	1.31	(0.67–2.59)	0.432
DM	0.21	(0.03–1.48)	0.117	0.20	(0.03–1.42)	0.107
CHD	1.11	(0.41–3.01)	0.837	0.89	(0.32–2.49)	0.830
Valvular heart disease	0.94	(0.35–2.55)	0.905	0.82	(0.29–2.31)	0.712
Cardiomyopathy	2.53	(1.11–5.77)	0.027	1.81	(0.76–4.34)	0.182
Stroke/TIA	0.75	(0.24–2.37)	0.630	0.85	(0.27–2.71)	0.781
OSAHS	0.24	(0.03–1.69)	0.150	0.16	(0.02–1.12)	0.065

Abbreviations: AF, atrial fibrillation; BMI, body mass index; CHD, coronary heart disease; DM, diabetes mellites; HF, heart failure; HTN, hypertension; LAD, left atrial diameter; LVEF, left ventricular ejection fraction; OSAHS, obstructive sleep apnea‐hypopnea syndrome; TIA, transient ischemic attack.

## 4 Discussion

The present study analyzed the clinical and procedural features, and follow‐up outcomes in young AF patients who underwent CA. Our findings showed that: (1) The prevalence of comorbidities was uncommon among young AF patients who underwent CA; (2) Following CA, AF symptoms exhibited significant improvement, with an overall recurrence rate of 14.4% observed during the 249‐day follow‐up period. Notably, patients with non‐paroxysmal AF manifested nearly double the recurrence rate compared to those with paroxysmal AF; (3) Non‐paroxysmal AF and HTN emerged as independent predictors of AF recurrence post‐CA among young AF patients.

Previous research has indicated that young patients with AF, aged 45 years or younger, constitute approximately 8.2% to 15% of the total AF patient population [8, 10]. The current study meticulously scrutinized the clinical attributes of 6,531 young AF patients who underwent single CA. The overarching characteristics of these young AF patients predominantly reflected a male composition, a notable prevalence of paroxysmal AF, lower CHA_2_DS_2_‐VASc scores, pronounced symptomatology, and a dearth of comorbidities. Notably, patients with non‐paroxysmal AF exhibited elevated values concerning age, BMI, duration of hospital stay, systolic blood pressure, heart rate, and LAD in comparison to those with paroxysmal AF. These unfavorable attributes of young AF patients may be linked to an increased occurrence of complications associated with non‐paroxysmal AF, such as heart failure, stroke/TIA, and cardiomyopathy. Furthermore, in contrast to non‐paroxysmal AF, paroxysmal AF tends to be less asymptomatic and displays a higher prevalence of HTN. So‐Ryoung et al. reported that young patients with grade 1 and grade 2 hypertension face an elevated risk of developing AF by 16%–25%, although they did not specify the impact of hypertension on the specific type of AF [11]. Young AF patients often present with significant clinical symptoms, exhibit suboptimal medication adherence, which significantly impacts their daily quality of life, and are more inclined to explore more efficacious treatment avenues [8].

Clinical studies and meta‐analyses have consistently demonstrated that CA surpasses antiarrhythmic drug therapy in not only reducing all‐cause rehospitalization but also enhancing the long‐term quality of life for patients [4, 12]. Regarding ablation characteristics, the majority of patients underwent PVI and successfully converted to sinus rhythm postablation, with few complications associated with CA procedures. Our study revealed a recurrence rate of 14.4% following a single ablation in young patients with AF, notably lower in paroxysmal AF compared to non‐paroxysmal AF, and an overall sinus rhythm maintenance rate of approximately 85.6%. The recurrence rate is significantly lower than that reported for elderly AF patients by Chadi et al. while the maintenance of sinus rhythm closely aligns with the findings reported by Saguner et al. [13, 14]. Compared to the general AF patients undergoing CA [15], this study demonstrates a notably low incidence of complications in young AF patients undergoing CA, with no statistical difference observed between paroxysmal and non‐paroxysmal AF. Moreover, EHRA scores indicate a notable improvement in symptoms among young AF patients after CA. Therefore, CA emerges as a safe and effective therapeutic approach for young AF patients, offering satisfactory atrial arrhythmia‐free survival and an acceptable complication rate.

The higher success rate observed in young patients undergoing ablation for AF may be attributed to several factors: (1) Disease duration and left atrial characteristics: Young patients with AF tend to have a shorter disease duration, smaller LAD, and less atrial electroanatomic remodeling and fibrosis compared to older patients. This results in a relatively uncomplicated atrial matrix, making the ablation procedure more effective. (2) Lower risk profile: Previous research has highlighted that combined cardiovascular and cerebrovascular diseases are significant risk factors for the progression from paroxysmal AF to persistent AF within a 5‐year period [6, 16]. In our study, young AF exhibited fewer comorbidities and a lower CHA_2_DS_2_‐VASc score, indicating a lower overall risk profile compared to older patients.

Although CA has emerged as an effective and safe therapeutic approach for patients with AF, the high recurrence rate remains a persistent challenge. Several risk factors contributing to AF recurrence post‐CA have been identified, including AF pattern and LA diameter [17, 18, 19]. However, there is a dearth of literature focused on young patients. Saguner et al. reported that obesity and structural heart disease independently predicted AF recurrence in young patients [13]. Similarly, Moran et al. identified hypertrophic cardiomyopathy as the sole predictor of recurrence in patients under 40 years old with AF [20]. Our findings revealed that non‐paroxysmal AF and HTN emerged as independent predictors of recurrence. Patients with non‐paroxysmal AF typically have a longer AF duration, larger LAD, and more pronounced atrial electroanatomic remodeling, factors that contribute to AF recurrence. Moreover, it is intuitive that persistent atrial arrhythmias may correlate with increased complication rates, underscoring the importance of early diagnosis and improved rhythm management for optimal AF outcomes [21]. The prevalence of hypertension in the young population has been steadily increasing over the years [22, 23], with approximately 50% of individuals aged 20 to 39 years in South Korea presenting with grade 1 or 2 hypertension [24]. Hypertension primarily affects the left atrium through hemodynamics and neurohormonal mechanisms, resulting in increased left atrial pressure, elevated release of atrial natriuretic peptide and angiotensin, leading to structural remodeling characterized by atrial fibrosis, electrical remodeling, Ca^2+^ handling remodeling, and pro‐inflammatory remodeling, ultimately culminating in impaired left atrial contraction function and AF [25, 26].

## 5 Limitations

This study is subject to several limitations. Firstly, our analysis categorized data solely based on paroxysmal AF and non‐paroxysmal AF, without further comparisons based on gender, age, AF recurrence and other relevant conditions. Nevertheless, we meticulously investigated the common clinical characteristics among young patients with AF. Secondly, variations in surgeons’ techniques may influence the outcomes of AF CA. However, given the superior atrial matrix quality in young AF patients compared to elderly patients, the ablation procedure tends to be straightforward, mitigating the impact of surgical disparities. Thirdly, being an observational study, potential confounding factors like differences in medication adherence, postablation lifestyle modifications, and intermittent monitoring of arrhythmia postablation may influence follow‐up results. Nonetheless, these factors are uniformly present across this study and do not substantially alter our conclusions.

## 6 Conclusion

Young AF patients undergoing CA exhibit fewer comorbidities, a low recurrence rate, and significant improvement in symptoms post‐CA. Non‐paroxysmal AF and HTN emerge as primary factors contributing to recurrence postablation among young AF patients.

## Conflicts of Interest

The authors declare no conflict of interest.

## Supporting information


**Figure S1.** EHRA score for symptom at baseline and follow‐up.

## Data Availability

The data sets used and analyzed during the current study are available from the corresponding author on reasonable request.
