# Supplementary figures and images for: Clinical Characteristics and Outcomes of Catheter Ablation in Young Patients With Atrial Fibrillation
Source: Clin Cardiol. 2025 May 13;48(5):e70144. doi: 10.1002/clc.70144 (PMC12070253; doi:10.1002/clc.70144)

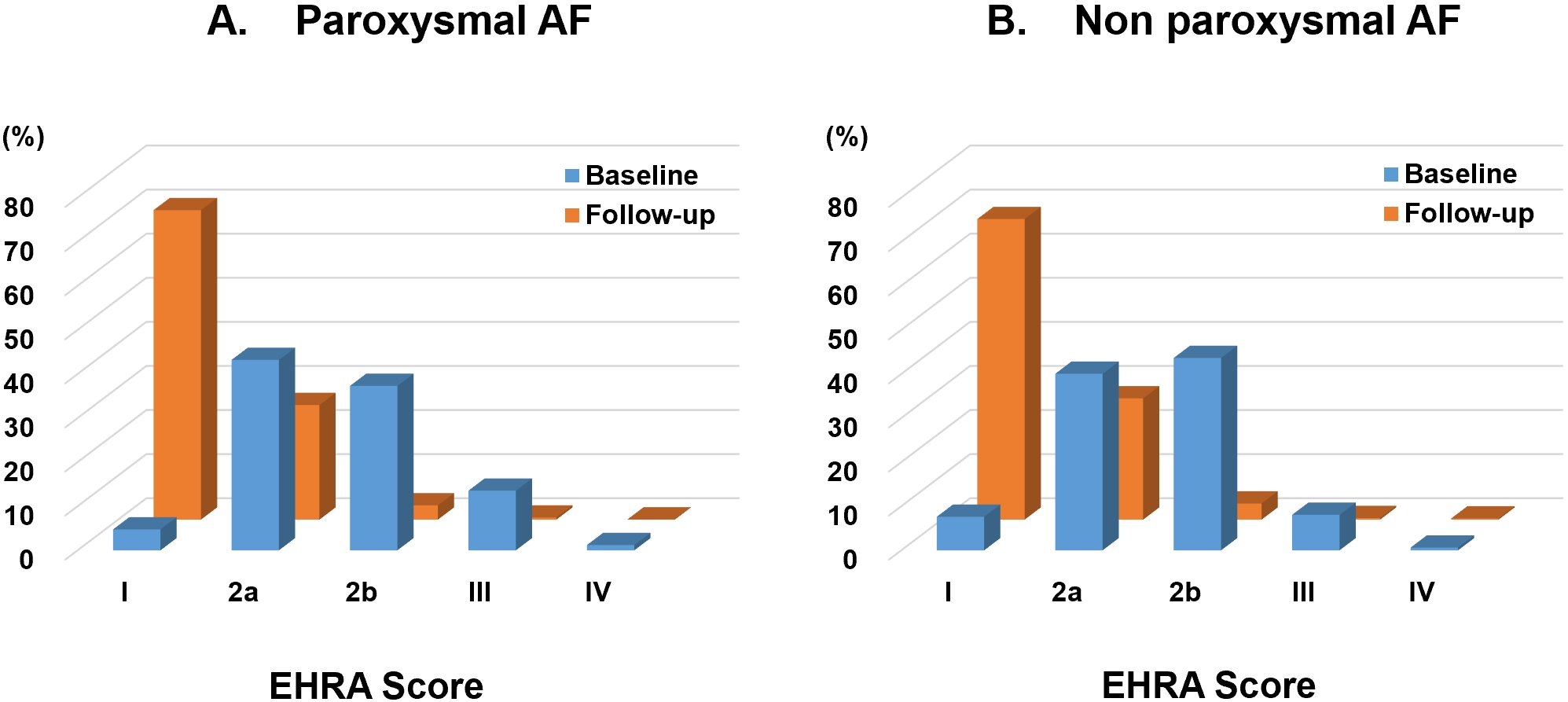

Supplement: Supplementary file 1 — Figure S1. EHRA score for symptom at baseline and follow‐up. [file CLC-48-e70144-s001.png]
